# Maternal experience of intimate partner violence and its association with morbidity and mortality of children: Evidence from India

**DOI:** 10.1371/journal.pone.0232454

**Published:** 2020-04-30

**Authors:** Pintu Paul, Dinabandhu Mondal

**Affiliations:** Centre for the Study of Regional Development, School of Social Sciences, Jawaharlal Nehru University, New Delhi, India; University of Botswana, BOTSWANA

## Abstract

**Objective:**

This study attempts to investigate the association between maternal exposure to intimate partner violence (IPV) and morbidity and mortality of children.

**Study design:**

A cross-sectional study was carried out using the most recent nationally representative data of the National Family Health Survey (NFHS-4) in India.

**Results:**

The prevalence of morbidity and mortality was higher among the children whose mothers faced physical, emotional, or sexual violence perpetrated by the partner than those who did not encounter any violence. Multivariate analysis revealed that maternal exposure to physical and sexual violence significantly increased the risks of childhood diarrhea and fever; and emotional violence was associated with an increased likelihood of diarrhea, fever, and acute respiratory infection (ARI) in the past 2 weeks among under-five children. Moreover, women’s experience of physical and emotional violence were associated with increased odds of infant mortality (<1 year) and under-five mortality (<5 years) in crude analysis. However, these associations were insignificant in the adjusted analysis. Similarly, we did not find any significant association between maternal exposure to IPV and child mortality (1 to < 5 years).

**Conclusion:**

Maternal experience of domestic violence was associated with an increased risk of childhood morbidity (diarrhea, fever and ARI). However, no significant association was found between violence against women and mortality of children. Prevention of domestic violence may help to reduce childhood illnesses. Additional efforts are needed for maternal and child healthcare programs to improve health status of women and children.

## Introduction

Child mortality remains one of the major public health concerns worldwide. Approximately 5.3 million children under age of 5 years died in 2018 globally. Among them, 2.5 million children died before completing 28 days of their life. Under-five mortality is highly prevalent in sub-Saharan Africa (2.8 million) and Central and Southern Asia (1.5 million), accounting for more than 80% of all under-five deaths, while these two regions constitute 52% of total under-five children [1, 2]. In 2018, approximately 882,000 deaths of under-five children are reported in India which is the highest among South Asian countries. In India, under-five mortality rate is also very high (37 per 1000 live births) [1].

Although 195 countries under Millennium Development Goals (MDGs)-4 aimed to reduce the under-five mortality by two-third during the period from 1990 to 2015, many countries especially low and lower-middle-income countries could not achieve the target. However, considerable progress has been made in reducing mortality of children, as 125 countries have already achieved the target of Sustainable Development Goals (SDGs) regarding under-five mortality of 25 or below per 1000 live births in 2018, and 21 countries are expected to do so by 2030. As per estimates, about 11 million reductions are required in under-five mortality in the rest of 53 countries which are falling behind to meet the target of SDGs on time [1, 3].

Morbidity and mortality of children aged 5 years or younger are determined by a complex interplay of various biological, socio-economic, and environmental conditions [4–7]. Poor nutritional status increases the risks of morbidity and mortality of children [8–9]. Similarly, Bryce et al. [10] noted that under-nutrition is an underlying reason for 53% deaths among children younger than 5 years of age. As per findings of The Million Death Study Collaborators [11] study, most of the children’s death in India occurs because of 5 main reasons: prematurity and low birth weight, neonatal infections, birth asphyxia and birth trauma, diarrheal diseases, and pneumonia.

Although major clinical and socio-economic causes of child mortality have been extensively examined, few studies have focused on the aspects related to social environment. The domestic violence against women is one such social determinant of health. It is reported that about 15–71% women face physical and/or sexual violence in their lifetime worldwide. Violence against women, which is manifested in a complex pattern of physical aggression, sexual coercion, emotional and psychological abuse, and controlling behaviors, bring harmful effects on a child’s health directly or indirectly [12, 13]. Previous studies indicated that mother’s exposure to violence aggravates the negative consequences on children’s nutritional status [14–16]. Violence and consequent mental stress of women further augment the vulnerabilities of abuse and maltreatment of children [17]. Similarly, exposure to parental violence directly affects the psychological health of children [18].

Maternal encounter with violence has severe implications on the morbidity and mortality of children as well; however, limited attention has been paid in this regard, especially in India. In a study of Bangladesh, Silverman et al. [19] observed that the prevalence of morbidity, for instance, diarrhea and acute respiratory infection (ARI), is much higher among the children whose mothers faced violence. Another study in this country found an association between consistent physical violence against women and child mortality [20]. Similar findings were also reported by studies in Africa (Uganda and Ethiopia) [21, 22], United States [23], and South Asia [24]. One of the limited studies in India by Ackerson and Subramanian [25] described that maternal experience of physical violence increases the risk of mortality among the children. However, their study only considered the physical violence against women perpetrated by the partner, and also the data used for the analysis is quite older, i.e., 2005–06. In this context, our study attempts to examine the association between exposure to IPV (physical, emotional, and sexual) of ever-married women aged 15–49 years and the morbidity and mortality of children below 5 years in India using the most recent data of National Family Health Survey, 2015–16.

## Methods

### Data source

The data from National Family Health Survey (NFHS-4) conducted in 2015–2016 were used in this study. The NFHS-4 is a nationally representative large-scale sample survey. The survey was carried out across the states and union territories of India, including as high as 601,509 households and 699,686 women aged 15–49 years with a response rate of 97%. The main objective of this survey was to collect updated and reliable information on various aspects of maternal and child health, such as fertility, mortality, family planning methods, utilization of maternal health care services, breastfeeding practices, nutritional status of mother and young children, child immunization, childhood morbidity and mortality, awareness and behavior regarding HIV/AIDS, and among others. The samples in NFHS-4 were selected using a two-stage stratified sampling design comprising of 28,586 clusters; 8,397 in urban, 20,059 in rural areas, and 130 slums. The slums were selected from the list provided by Municipal Corporation Offices (MCOs). In the first stage, the clusters were selected using the method of probability proportional to size (PPS). In the second stage, a complete household mapping and listing was done in the selected cluster and 22 households were randomly chosen in each cluster in a systematic manner from the household listing. The sampling frame used in this survey was the 2011 Indian Population and Housing Census. A detailed description of the sampling design and survey procedure is provided in the NFHS-4 national report [26].

### Study participants

**Fig 1:**
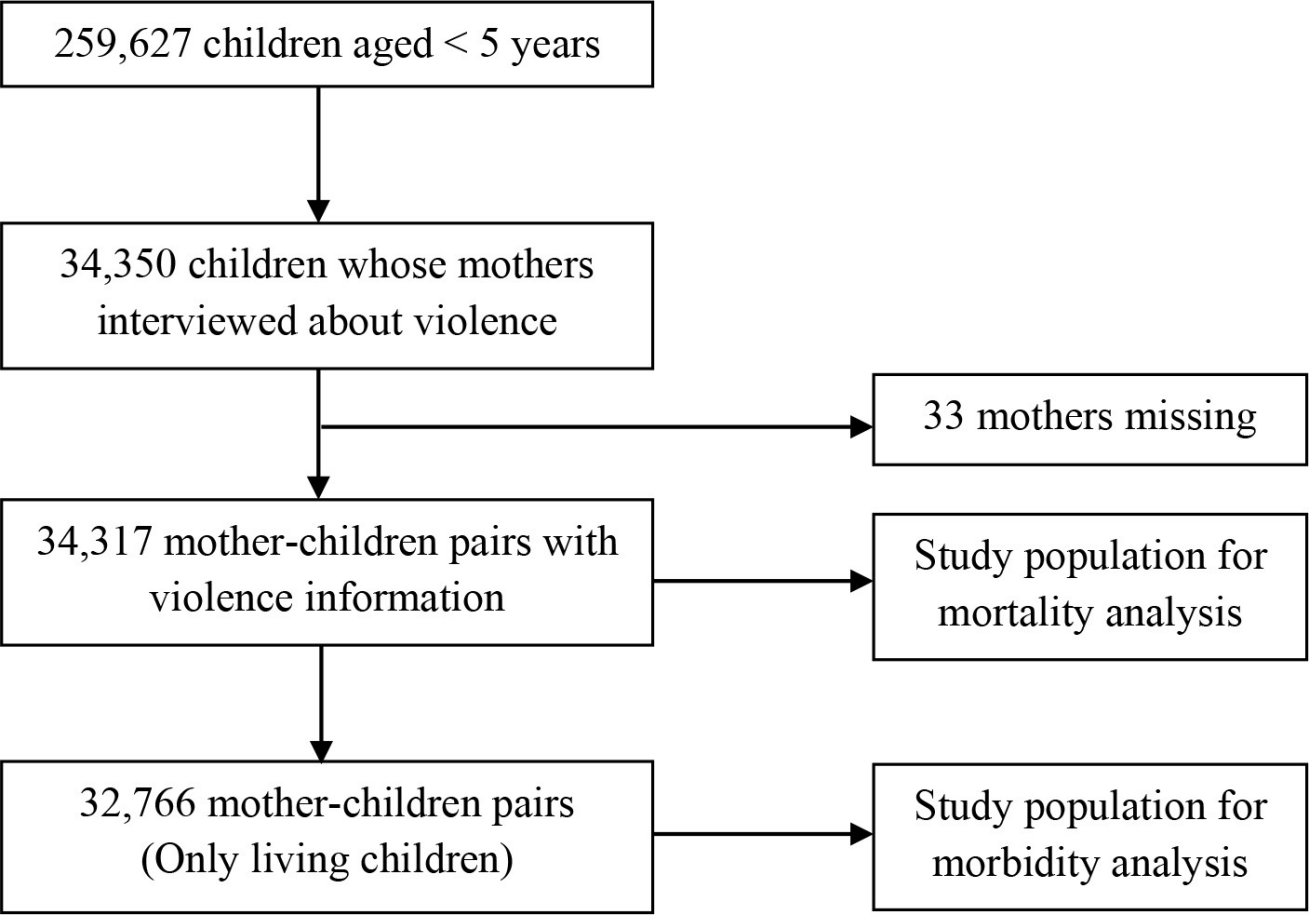
Flow chat for the selection of study participants, NFHS-4 (2015–16).

The NFHS-4 provided information for 259,627 children born to the women aged 15–49 years in the past 5 years preceding the survey. Out of these children, 34,350 had mothers who had been selected and interviewed regarding domestic violence at the time of the survey. However, complete information about violence was available for 34,317 mother-children pairs, which have been considered for mortality analysis. Of these, 32,766 living children were included for morbidity analysis. Additionally, there were some missing cases in morbidity variables (i.e., diarrhea and fever) (Fig 1).

### Outcome variables

The outcome variables of this study are morbidity and mortality of children below 5 years of age. Children’s morbidity was assessed with 3 variables: diarrhea, fever, and ARI in the past 2 weeks. For each child under 5 years, the mothers were asked whether the child had illness from diarrhea or fever in the past 2 weeks prior to the survey. ARI was defined as cough accompanied with short, rapid or difficult breathing that is chest related. The mortality indicators in this study include infant mortality, child mortality, and under-five mortality. Infant mortality was defined as death of children below 12 months of age. Similarly, child and under-five mortality were measured by the death of children between 1 to 5 years and below 5 years of age, respectively. All 6 outcome variables related to morbidity and mortality of children were dichotomized into binary responses (0 and 1).

### Predictor variables

Maternal exposure to IPV is the main predictor variable in this study. Information about violence was obtained from the ever-married women during the survey of NFHS-4. To extract maximum information regarding violence committed by the current husband or the most recent husband, a sequence of questions were asked to the women. Three major types of violence–physical, emotional, and sexual–were identified in this survey. Each woman selected for the violence module were asked whether her husband has ever 1) pushed, shook, or thrown something at her, 2) slapped her, 3) twisted her arm or pulled her hair, 4) punched her with his fist or with something that could hurt her, 5) kicked, dragged, or beaten her up, 6) tried to choke or burn her on purpose, or 7) threaten or attacked with a knife, gun, or any other weapon as components of physical violence. Sexual violence includes whether the women were: 1) physically forced to have sexual intercourse, 2) physically forced to perform any other sexual acts, or 3) forced with threats or in any other way to perform sexual acts when she did not want to. Similarly, emotional violence against women by the husband was judged by 3 questions in particular, such as, 1) humiliated in front of others, 2) threatened to hurt or harm, or 3) insulted or made her feel bad about herself. The questions and procedures related to violence against women have been illustrated in detail in the national NFHS-4 report [26]. Using this detailed recorded information, 3 binary variables were created about violence that includes whether the women experience any physical violence, sexual violence, or emotional violence. For the purpose of this study, a final variable of women’s exposure to violence was developed with 5 responses: 1) experience of any physical violence, 2) experience of any sexual violence, 3) experience of any emotional violence, 4) experience of all three forms of violence and 5) experience no violence from her current or former husband.

### Covariates

Besides the exposure to violence, some important confounding variables were also included in this study that could potentially influence a child’s morbidity and mortality. These factors are related to child and maternal demographics and socio-economic characteristics. Demographics of child comprise 3 variables, age of child (0–11, 12–35 and 36–59 months), sex of child (male and female) and birth order (<3 and ≥3) which are important factors for child mortality and morbidity [27]. Similarly, maternal demographics include age of the mother (15–24, 25–34 and 35–49 years) and age at marriage (<18 and ≥18 years). Mother’s education which is an important factor for determining child morbidity and mortality was categorized into 4 groups: no education, primary, secondary, and higher education. Father’s education status was also included accordingly. Maternal body mass index (BMI) and anaemia are two vital elements that measures mother’s nutritional status and may also bring significant effects on children’s health. BMI was assessed using World Health Organization (WHO) standards as <18.5 kg/m^2^ (underweight), 18.5 to 24.9 kg/m^2^ (normal), and ≥25.0 kg/m^2^ (overweight). Further, anaemia was measured from the haemoglobin level; if the level of haemoglobin in the blood is below 11.0 g/dl, the mother is considered as anaemic [26]. Furthermore, the socio-economic factors, such as social group, religion, place of residence, and household wealth were taken into consideration. Social group plays an important role in children’s morbidity and mortality occurrence. In this study, social group was divided into three categories as Scheduled Caste (SC)/Scheduled Tribe (ST), Other Backward Classes (OBC) and others (i.e., General). Religious affiliation is also considered as a confounding variable to determine the morbidity and mortality. Religion was categorized into Hindu, Muslim and others. Place of residence was included to observe rural and urban differences. Household wealth index was estimated from the ownership of household assets, housing characteristics, and access to drinking water and sanitation facilities. A score was generated for each individual using principal component analysis (PCA) and then grouped into 5 quintiles, each represents 20% of the total respondents, between 1 (poorest) and 5 (richest).

### Statistical analyses

Univariate statistics were carried out to describe the distribution of key predictors, confounders, and outcomes variables. Bivariate percentage distribution was estimated to assess the differences in the morbidity and mortality of children by predictor variables. The sample weight was used for estimation of percentage distribution. Detail descriptions of sample weight have been provided in the national report of NFHS-4 [26]. Finally, binary logistic regression models were employed to examine the crude and adjusted association between the violence against women and the morbidity and mortality of children. The regression results were presented by crude and adjusted odds ratio (aOR) with 95% confidence interval (CI). All the statistical analyses were performed using STATA version 14.0 (StataCorp LP, College Station, TX, USA).

## Results

### Participant’s characteristics

Among total study participants (n = 34,317), approximately 29%, 13%, and 7% women reported experiences of physical, emotional, and sexual violence, respectively. Additionally, 3.5% women were exposed to all forms of domestic violence. Among the total under-five children born to women aged 15–49 years, one-firth (19%) were infants, 52% were male, and 68% had birth order below 3. Over half of the women (58%) belonged to age group of 25–34 years and 37% were married before 18 years. About 29% women were illiterate, while 17% of the partner had no formal education. About 24% mothers were underweight and 56% were anaemic. Majority of the respondents were living in rural areas (73%), belonged to OBC (42%), believed in Hindu religion (73%), and from the poorest (24%) and poorer (22%) wealth quintile group (Table 1).

**Table 1:** Descriptive statistics of key predictors, socio-demographic characteristics and outcome variables.

Variables	Frequency (n)	Percentage (%)
**Key predictors**		
Experience physical violence		
No	24,159	71.4
Yes	10,158	28.6
Experience emotional violence		
No	30,516	89.0
Yes	4,354	12.6
Experience sexual violence		
No	31,809	92.6
Yes	2,508	7.4
Experience all forms of violence		
No	33,115	96.5
Yes	1,202	3.5
**Control variables**		
Age of child in months		
0–11	6,208	19.2
12–35	13,606	40.1
36–59	14,503	40.7
Sex of child		
Male	17,863	52.0
Female	16,454	48.0
Birth order		
<3	22,482	68.2
≥3	11,835	31.8
Women's age in years		
15–24	9,778	31.4
25–34	20,908	58.3
35–49	3,631	10.4
Age at marriage in years		
<18	12,461	37.2
≥18	21,284	62.8
Women's education		
No education	10,686	29.3
Primary	4,986	13.9
Secondary	15,395	46.2
Higher	3,250	10.6
Father's education		
No education	6,505	17.4
Primary	5,230	14.8
Secondary	18,374	54.5
Higher	4,117	13.3
Maternal BMI		
Underweight	8,005	23.8
Normal	20,881	61.0
Overweight	4,947	15.2
Maternal Anaemia		
Not anaemic	14,318	43.6
Anaemic	19,317	56.4
Place of residence		
Urban	8,488	27.4
Rural	25,829	72.6
Social group		
SC/ST	13,522	38.0
OBC	13,193	42.2
Others	5,927	19.8
Religion		
Hindu	24,582	72.2
Muslim	5,562	16.8
Others	4,173	11.0
Wealth quintile		
Poorest	9,123	24.2
Poorer	8,084	22.4
Middle	6,917	19.8
Richer	5,600	17.6
Richest	4,593	16.0
**Outcome variables**		
Diarrhea in past 2 weeks		
No	29,669	90.7
Yes	3,036	9.3
Fever in past 2 weeks		
No	28,365	86.6
Yes	4,347	13.4
ARI in past 2 weeks		
No	31,832	97.2
Yes	934	2.8
Infant mortality		
No	32,927	96.0
Yes	1,390	4.1
Child mortality		
No	34,156	99.6
Yes	161	0.4
Under-five mortality		
No	32,766	95.5
Yes	1,551	4.5

Percentages are computed from weighted sample.

Among the living children born in the past 5 years (n = 32,766), 9.3% had diarrhea, 13.4% had fever and 2.8% had ARI in the past 2 weeks before the survey. Among total sample children (n = 34,317), 4.5% died before celebrating their fifth birthday; where most of them (4.1%) died before completing one year of age (Table 1).

### Child morbidity and mortality by predictors and confounders

The prevalence of morbidity and mortality was substantially higher among the children whose mothers experienced physical, emotional or sexual violence compared to those who were not exposed to any form of domestic violence. The incidence of morbidity was higher among the infants than the older children. Moreover, childhood illnesses were more common among males and children born with 3 or higher birth order. Corresponding to this result, the occurrence of mortality was also higher among male and high parity children. Morbidity was higher among the children of younger mothers than older ones. However, the indicators of mortality appeared to be worse among older women. Child married women reported higher cases of children’s diarrhea and mortality compared to those who got married as adults. The prevalence of morbidity and mortality was higher among the children of parents who had the below secondary level of education. Underweight women reported higher proportion of mortality and diarrhea among children. Contrastingly, fever had a U-shaped distribution with maternal BMI as fever being higher among children of both underweight and overweight women. Childhood morbidity was higher among anaemic women than those who were not anaemic. Children belonged to Muslim and OBC were exposed to higher illness than the other groups. Additionally, a considerably higher proportion of rural and poor children reported illnesses and mortality in the past 2 weeks (Table 2).

**Table 2:** Percentage distribution of child morbidity and mortality by explanatory variables.

Variables	Morbidity indicators			Mortality indicators		
	Diarrhea	Fever	ARI	Infant mortality (< 1 year)	Child mortality (1 to < 5 years)	Under-five mortality (< 5 years)
Experience physical violence						
No	8.3	12.4	2.5	3.8	0.4	4.2
Yes	11.7	15.8	3.5	4.6	0.6	5.2
Experience emotional violence						
No	8.7	12.6	2.5	4.0	0.4	4.4
Yes	13.4	18.5	4.9	4.6	0.8	5.3
Experience sexual violence						
No	8.9	13.0	2.7	4.0	0.4	4.4
Yes	13.6	17.4	4.2	5.0	0.5	5.5
Experience all forms of violence						
No	9.1	13.1	2.7	4.0	0.4	4.4
Yes	15.4	19.8	5.6	5.1	0.6	5.7
Age of child in months						
0–11	14.1	14.7	3.6	–	–	–
12–35	11.1	15.3	2.8	–	–	–
36–59	5.2	10.8	2.4	–	–	–
Sex of child						
Male	9.7	14.1	3.2	4.4	0.4	4.8
Female	8.8	12.6	2.4	3.7	0.5	4.2
Birth order						
<3	9.1	12.7	2.8	3.7	0.3	3.9
≥3	9.7	14.8	2.9	4.9	0.8	5.6
Women's age in years						
15–24	10.7	13.5	3.1	4.7	0.3	4.9
25–34	8.9	13.4	2.8	3.6	0.4	4.0
35–49	7.2	12.6	2.2	4.9	0.7	5.6
Age at marriage in years						
<18	9.6	13.2	2.8	4.4	0.6	5.0
≥18	9.1	13.5	2.8	3.8	0.3	4.2
Women's education						
No education	9.4	12.7	2.8	5.6	0.7	6.3
Primary	10.0	14.8	2.7	4.9	0.5	5.4
Secondary	9.3	13.7	2.8	3.1	0.3	3.4
Higher	7.8	12.2	3.1	2.9	0.2	3.1
Father's education						
No education	9.5	12.3	3.0	5.7	0.9	6.6
Primary	9.7	14.3	2.5	4.8	0.5	5.2
Secondary	9.3	13.6	2.8	3.7	0.3	4.1
Higher	8.3	12.9	2.7	2.4	0.1	2.5
Maternal BMI						
Underweight	9.9	13.9	2.8	4.3	0.6	4.9
Normal	9.3	12.7	3.0	4.2	0.4	4.6
Overweight	8.5	15.2	2.2	3.3	0.3	3.5
Maternal Anaemia						
Not anaemic	8.6	13.1	2.7	4.1	0.4	4.5
Anaemic	9.9	13.5	2.9	4.0	0.4	4.5
Place of residence						
Urban	9.0	12.8	2.4	3.4	0.2	3.6
Rural	9.4	13.6	2.9	4.3	0.5	4.8
Religion						
Hindu	9.2	12.8	2.5	4.2	0.5	4.7
Muslim	10.8	16.9	4.1	4.0	0.3	4.3
Others	7.8	12.0	3.0	3.4	0.3	3.6
Social group						
SC/ST	9.0	12.4	2.8	4.2	0.5	4.7
OBC	10.5	14.2	2.7	4.4	0.5	4.9
Others	8.0	13.4	2.9	3.1	0.2	3.2
Wealth index						
Poorest	10.1	13.2	3.0	5.2	0.8	6.0
Poorer	9.3	13.9	3.1	4.8	0.4	5.3
Middle	9.3	13.5	2.8	3.8	0.4	4.2
Richer	9.2	13.3	2.7	3.5	0.2	3.7
Richest	8.1	12.9	2.2	2.3	0.1	2.4

Percentages are computed from weighted sample.

### Association between maternal experience of IPV and morbidity and mortality of children

Crude analysis revealed that maternal experience of physical, emotional and sexual violence was associated with increased odds of diarrhea, fever and ARI in past 2 weeks among under-five children. Multivariate analysis of this study indicated that children whose mothers experienced physical (aOR: 1.34, 95% CI: 1.21–1.49), emotional (aOR: 1.64, 95% CI: 1.45–1.86), sexual (aOR: 1.30, 95% CI: 1.07–1.59) or all forms of violence (aOR: 2.08, 95% CI: 1.74–2.46) were more likely to have diarrhea than those who did not encounter any spousal violence. Women exposed to physical (aOR: 1.10, 95% CI: 1.00–1.21), emotional (aOR: 1.49, 95% CI: 1.33–1.67), sexual (aOR: 1.32, 95% CI: 1.11–1.56) or all forms of violence (aOR: 1.82, 95% CI: 1.55–2.14) were significantly associated with an increased likelihood of fever in the past 2 weeks among under-five children. The likelihood of children’s ARI was higher among the women who were emotionally abused (aOR: 1.68, 95% CI: 1.36–2.08) or experienced all forms of violence (aOR: 1.93, 95% CI: 1.42–2.61) perpetrated by the partner (Table 3).

**Table 3:** Association between maternal experience of intimate partner violence (IPV) and childhood morbidity.

Maternal exposure to violence	Diarrhea in past 2 weeks		Fever in past 2 weeks		ARI in past 2 weeks	
	cOR (95% CI)	aOR (95% CI)	cOR (95% CI)	aOR (95% CI)	cOR (95% CI)	aOR (95% CI)
No violence ®	1.00	1.00	1.00	1.00	1.00	1.00
Physical violence	1.33 (1.20–1.46)†	1.34 (1.21–1.49)†	1.08 (0.99–1.18)	1.10 (1.00–1.21)*	0.98 (0.81–1.18)	1.03 (0.84–1.25)
Emotional violence	1.63 (1.44–1.84)†	1.64 (1.45–1.86)†	1.45 (1.30–1.61) †	1.49 (1.33–1.67) †	1.69 (1.39–2.06)†	1.68 (1.36–2.08)†
Sexual violence	1.30 (1.08–1.58)†	1.30 (1.07–1.59)†	1.27 (1.08–1.49) †	1.32 (1.11–1.56) †	1.04 (0.74–1.48)	1.06 (0.73–1.53)
All forms of violence	2.07 (1.75–2.45)†	2.08 (1.74–2.46)†	1.74 (1.50–2.03) †	1.82 (1.55–2.14) †	1.90 (1.43–2.52)†	1.93 (1.42–2.61)†

® Reference category

† *p*<0.01

* *p*<0.05

*cOR* crude odds ratio; *aOR*, adjusted odds ratio; *CI*, confidence interval

Adjusted models are controlled for age of child, sex of child, birth order, age of mother, maternal age at marriage, maternal education, father’s education, maternal BMI, maternal anaemia, place of residence, caste, religion, and wealth quintile.

Furthermore, women’s victimization of physical, emotional, or all forms of violence was associated with an increased likelihood of infant mortality (<1 year) and under-five mortality (<5 years) in crude analysis. However, these associations became insignificant after inclusion of important confounders in the adjusted models. However, we did not find any significant association between women’s exposure to IPV and child mortality (1 to <5 years) (Table 4).

**Table 4:** Association between maternal experience of intimate partner violence (IPV) and mortality of children.

Maternal Exposure to Violence	Infant mortality (died in <1 year)		Child mortality (died in 1- <5 years)		Under-five mortality (died in <5 years)	
	cOR (95% CI)	aOR (95% CI)	cOR (95% CI)	aOR (95% CI)	cOR (95% CI)	aOR (95% CI)
No violence ®	1.00	1.00	1.00	1.00	1.00	1.oo
Physical violence	1.25 (1.09–1.44)†	1.07 (0.92–1.24)	1.33 (0.89–1.98)	1.07 (0.71–1.62)	1.26 (1.10–1.44)†	1.07 (0.93–1.23)
Emotional violence	1.23 (1.02–1.47)*	1.01 (0.83–1.23)	1.47 (0.90–2.41)	1.28 (0.77–2.11)	1.26 (1.06–1.49)†	1.04 (0.86–1.25)
Sexual violence	1.20 (0.91–1.57)	0.98 (0.73–1.30)	0.91 (0.37–2.23)	0.73 (0.29–1.80)	1.17 (0.90–1.51)	0.95 (1.72–1.25)
All forms of violence	1.47 (1.13–1.90)†	1.17 (0.89–1.54)	1.58 (0.77–3.26)	1.15 (0.55–2.40)	1.49 (1.16–1.90)†	1.17 (0.91–1.51)

® Reference category

† *p*<0.01

* *p*<0.05

*cOR*, crude odds ratio; *aOR*, adjusted odds ratio; *CI*, confidence interval

Adjusted models are controlled for sex of child, birth order, age of mother, maternal age at marriage, maternal education, father’s education, maternal BMI, maternal anaemia, place of residence, caste, religion, and wealth quintile.

## Discussion

This study provides important insights into the association between maternal experience of domestic violence and the morbidity and mortality of children. It is observed that a substantial proportion of children reported illnesses in the past 2 weeks preceding the survey and died under the age of 5 years at the time of survey. Moreover, women’s encounter with violence by their partner was also unacceptably high in India. The findings of this study indicate that maternal exposure to violence was significantly associated with increased risks of diarrhea, fever, and ARI in the past 2 weeks among under-five children. This finding is consistent with several other previous studies conducted in low- and middle-income countries. For instance, a study conducted in Uganda [21] reported that women’s lifetime experience of violence increased the risks of childhood illnesses. The findings also indicate two-fold increase in diarrhea among the children of abused mothers even after adjusting for education of mother, parity, residence, and age of children [21]. Similarly, a study carried out in 3 South Asian countries—Bangladesh, India and Nepal found that maternal experience of physical, sexual, or both forms of violence increased the risks of fever, diarrhea, and ARI of children in the past 2 weeks [24]. A prospective cohort study from rural Bangladesh also documented that the likelihood of children’s diarrhea and other illnesses was higher among the children of mothers who were exposed to different forms of domestic violence [28]. Another study conducted in Bangladesh using nationally representative samples illustrated that women’s past year experience of spousal violence raised the vulnerabilities of children’s ARI by 37% and diarrhea by 65% even after controlling for potential confounders [19].

Our findings also signify that maternal exposure to physical or emotional violence was associated with an increased likelihood of infant and under-five mortality in crude analysis. However, these associations were not significant after adjusting for child and maternal demographics and socio-economic background factors. These findings are in the same line of earlier studies conducted in India [25, 29–32]. Silverman et al. [32] also found that the chances of infant and under-five mortality were greater among girls than boys born to mothers experiencing spousal violence. The findings of the present study also confirmed the results found in Bangladesh [20]. Similarly, a study carried out in 5 African countries reported that physical violence against women was strongly associated with mortality (<2 years) in Kenya. However, the association was weak in Egypt, Honduras and Malawi. Moreover, maternal experience of sexual violence had an increased likelihood of mortality in Malawi [15]. This finding is in contrast with our present study. In our study, women’s exposure to sexual violence had no significant association with mortality of children.

Maternal exposure to domestic violence perpetrated by the partner may directly or indirectly affect children’s health status. Studies indicated that domestic violence exerts a negative impact on the physical and psychological health of women [33]. Violence during pregnancy increases the risk of obstetric complications [34]. Moreover, physiological stress, anxiety and depression of abused women lead to lower utilization of maternity care and poor nutrition during pregnancy that may eventually result in premature birth or low birth weight babies [35]. It is evident that premature birth and low birth weight are found to be the primary causes of early childhood illnesses and mortality [7, 11]. Additionally, psychological stress of women is a risk factor for zinc and iron deficiencies [36, 37]. There are also studies indicating that mothers, who consistently experience psychological and physical aggression perpetrated by the partner, are at greater risks of anxiety and post-traumatic stress disorders which further results in maltreatment among their children [17]. Studies found a strong association between controlling behavior of husband and domestic violence against women [38, 39]. Moreover, controlling nature of husband may also lead to lower decision-making power of women. Lack of autonomy restricts women from seeking adequate healthcare facilities. Therefore, women’s inability to make decisions for healthcare imprints serious implications on their children’s health. Exposure to maternal violence also brings psychological stress on the children which further increases the risk of illnesses [40].

Additionally, our present study reported high incidence of infant and under-five mortality. It is also found that the episodes of diarrhea, fever and ARI were pervasive among disadvantaged socio-economic groups, which may contribute to a large number of deaths among under-five children in India. These findings, therefore, suggest for strengthening the existing maternal and child healthcare programs to combat childhood morbidity and mortality.

The findings of this study should be considered in light of some limitations. This study was unable to explain the possible mechanism driving the influence of women’s exposure to domestic violence on child morbidity and mortality because of cross-sectional study design. Further research is needed using longitudinal data to explore potential pathways causing high morbidity and mortality of children born to abused mothers. There could be under-reporting of domestic violence due to social stigma, privacy concerns, and sensitive nature of information [35]. Moreover, Indian society is by and large patriarchal; therefore, justification of wife-beating is substantially high [41]. Recall bias might have been introduced because women were asked to provide retrospective information regarding their experience of domestic violence. Furthermore, we could not include other important covariates in the analysis that could potentially impact on the morbidity and mortality of children due to the paucity of data. Despite these limitations, the present study provides important shreds of evidence for policy-making on the issues of domestic violence against women and children’s healthcare in India.

## Conclusion

The findings of this study indicate that maternal experience of physical and sexual violence was associated with increased risks of diarrhea and fever among under-five children. Moreover, women’s encounter with emotional violence was significantly associated with an increased likelihood of diarrhea, fever and ARI in children. However, mortality indicators of children had no significant association with domestic violence perpetrated by the partner. Policy-makers and stakeholders should intervene to prevent violence against women, which may help in reducing the risks of childhood illnesses. Additional efforts are needed for maternal and child healthcare programs to improve the health status of women and children.

## References

[1] UNICEF. Levels and Trends in Child Mortality: Report 2019 Estimates developed by the UN Inter-agency Group for child mortality estimation. New York: United Nations Children’s Fund; 2019 Available from https://childmortality.org/wp-content/uploads/2019/10/UN-IGME-Child-Mortality-Report-2019.pdf.

[2] United Nations. World Population Prospects 2019. New York: United Nations, Department of Economic and Social Affairs, Population Division; 2019 Available from https://population.un.org/wpp/Publications/Files/WPP2019_Highlights.pdf.

[3] You D, Hug L, Ejdemyr S, Idele P, Hogan D, Mathers C, et al Global, regional, and national levels and trends in under-5 mortality between 1990 and 2015, with scenario-based projections to 2030: a systematic analysis by the UN Inter-agency Group for Child Mortality Estimation. The Lancet. 2015;386(10010):2275–86. 10.1016/S0140-6736(15)00120-8 26361942

[4] Mølbak K, Aaby P, Ingholt L, Højlyng N, Gottschau A, Andersen H, et al Persistent and acute diarrhoea as the leading causes of child mortality in urban Guinea Bissau. Transactions of the Royal Society of Tropical Medicine and Hygiene. 1992;86(2):216–20. 10.1016/0035-9203(92)90580-6 1440794

[5] Farmer P. Social inequalities and emerging infectious diseases. Emerging infectious diseases. 1996;2(4):259 10.3201/eid0204.960402 8969243 PMC2639930

[6] Eisenberg JN, Desai MA, Levy K, Bates SJ, Liang S, Naumoff K, et al Environmental determinants of infectious disease: a framework for tracking causal links and guiding public health research. Environmental Health Perspectives. 2007;115(8):1216–23. 10.1289/ehp.9806 17687450 PMC1940110

[7] Liu L, Chu Y, Oza S, Hogan D, Perin J, Bassani DG, et al National, regional, and state-level all-cause and cause-specific under-5 mortality in India in 2000–15: a systematic analysis with implications for the Sustainable Development Goals. The Lancet Global Health. 2019;7(6):e721–34. 10.1016/S2214-109X(19)30080-4 31097276 PMC6527517

[8] Beau JP, Garenne M, Diop B, Briend A, Mar ID. Diarrhoea and nutritional status as risk factors of child mortality in a Dakar hospital (Senegal). Journal of tropical pediatrics. 1987;33(1):4–9. 10.1093/tropej/33.1.4 3573134

[9] Pelletier DL, Frongillo EA Jr, Schroeder DG, Habicht JP. The effects of malnutrition on child mortality in developing countries. Bulletin of the World Health Organization. 1995;73(4):443 7554015 PMC2486780

[10] Bryce J, Boschi-Pinto C, Shibuya K, Black RE, WHO Child Health Epidemiology Reference Group. WHO estimates of the causes of death in children. The Lancet. 2005;365(9465):1147–52. 10.1016/S0140-6736(05)71877-8 15794969

[11] Million Death Study Collaborators. Causes of neonatal and child mortality in India: a nationally representative mortality survey. The Lancet. 2010;376(9755):1853–60. 10.1016/S0140-6736(10)61461-4 PMC3042727 21075444

[12] World Health Organization (2005). WHO multi-country study on women’s health and domestic violence against women: summary report of initial results on prevalence, health outcomes and women’s responses. Geneva: World Health Organization Retrieved from: https://www.who.int/gender/violence/who_multicountry_study/summary_report/summary_report_English2.pdf

[13] Garcia-Moreno C, Jansen HA, Ellsberg M, Heise L, Watts CH. Prevalence of intimate partner violence: findings from the WHO multi-country study on women's health and domestic violence. The lancet. 2006;368(9543):1260–9. 10.1016/S0140-6736(06)69523-8 17027732

[14] Ackerson LK, Subramanian SV. Domestic violence and chronic malnutrition among women and children in India. American journal of epidemiology. 2008;167(10):1188–96. 10.1093/aje/kwn049 18367471 PMC2789268

[15] Rico E, Fenn B, Abramsky T, Watts C. Associations between maternal experiences of intimate partner violence and child nutrition and mortality: findings from Demographic and Health Surveys in Egypt, Honduras, Kenya, Malawi and Rwanda. Journal of epidemiology & community health. 2011;65(4):360–7. 20841374 10.1136/jech.2008.081810

[16] Ziaei S, Naved RT, Ekström EC. Women's exposure to intimate partner violence and child malnutrition: findings from demographic and health surveys in Bangladesh. Maternal & child nutrition. 2014;10(3):347–59. 22906219 10.1111/j.1740-8709.2012.00432.x PMC6860329

[17] Taylor CA, Guterman NB, Lee SJ, Rathouz PJ. Intimate partner violence, maternal stress, nativity, and risk for maternal maltreatment of young children. American journal of public health. 2009;99(1):175–83. 10.2105/AJPH.2007.126722 19008518 PMC2636621

[18] Robbie Rossman BB, Ho J. Posttraumatic response and children exposed to parental violence. Journal of Aggression, Maltreatment & Trauma. 2000;3(1):85–106.

[19] Silverman JG, Decker MR, Gupta J, Kapur N, Raj A, Naved RT. Maternal experiences of intimate partner violence and child morbidity in Bangladesh: evidence from a national Bangladeshi sample. Archives of pediatrics & adolescent medicine. 2009;163(8):700–5. 19652100 10.1001/archpediatrics.2009.115 PMC4456175

[20] Åsling‐Monemi K, Tabassum Naved R, Persson LÅ. Violence against women and the risk of under‐five mortality: analysis of community‐based data from rural Bangladesh. Acta Paediatrica. 2008;97(2):226–32. 10.1111/j.1651-2227.2007.00597.x 18254912

[21] Karamagi CA, Tumwine JK, Tylleskar T, Heggenhougen K. Intimate partner violence and infant morbidity: evidence of an association from a population-based study in eastern Uganda in 2003. BMC pediatrics. 2007;7(1):34. 17988374 10.1186/1471-2431-7-34 PMC2186330

[22] Garoma S, Fantahun M, Worku A. Maternal intimate partner violence victimization and under-five children mortality in Western Ethiopia: A case–control study. Journal of tropical pediatrics. 2012;58(6):467–74. 10.1093/tropej/fms018 22588551

[23] Wright RJ, Mitchell H, Visness CM, Cohen S, Stout J, Evans R, et al Community violence and asthma morbidity: the Inner-City Asthma Study. American journal of public health. 2004;94(4):625–32. 10.2105/ajph.94.4.625 15054016 PMC1448309

[24] Ferdousy EZ, Matin MA. Association between intimate partner violence and child morbidity in South Asia. Journal of Health, Population and Nutrition. 2015;33(1):16. 10.1186/s41043-015-0016-y PMC5025981 26825360

[25] Ackerson LK, Subramanian SV. Intimate partner violence and death among infants and children in India. Pediatrics. 2009;124(5):e878–89. 10.1542/peds.2009-0524 19822588

[26] International Institute for Population Sciences (IIPS), ICF. National Family Health Survey (NFHS-4), 2015–16. Mumbai: IIPS; 2019 Available from http://rchiips.org/NFHS/NFHS-4Reports/India.pdf.

[27] Kumar PP, File G. Infant and child mortality in Ethiopia: a statistical analysis approach. Ethiopian Journal of Education and Sciences. 2010;5(2).

[28] Åsling-Monemi K, Naved RT, Persson LÅ. Violence against women and increases in the risk of diarrheal disease and respiratory tract infections in infancy: a prospective cohort study in Bangladesh. Archives of pediatrics & adolescent medicine. 2009;163(10):931–6. 19805712 10.1001/archpediatrics.2009.167

[29] Jejeebhoy SJ. Associations between wife-beating and fetal and infant death: impressions from a survey in rural India. Studies in family planning. 1998:300–8. 9789323

[30] Ahmed S, Koenig MA, Stephenson R. Effects of domestic violence on perinatal and early-childhood mortality: evidence from north India. American journal of public health. 2006;96(8):1423–8. 10.2105/AJPH.2005.066316 16809594 PMC1522123

[31] Koenig MA, Stephenson R, Acharya R, Barrick L, Ahmed S, Hindin M. Domestic violence and early childhood mortality in rural India: evidence from prospective data. International journal of epidemiology. 2010;39(3):825–33. 10.1093/ije/dyq066 20444839 PMC2912486

[32] Silverman JG, Decker MR, Cheng DM, Wirth K, Saggurti N, McCauley HL, et al Gender-based disparities in infant and child mortality based on maternal exposure to spousal violence: the heavy burden borne by Indian girls. Archives of pediatrics & adolescent medicine. 2011;165(1):22–7. 21199976 10.1001/archpediatrics.2010.261 PMC3940346

[33] Campbell JC. Health consequences of intimate partner violence. The lancet. 2002;359(9314):1331–6. 10.1016/S0140-6736(02)08336-8 11965295

[34] Ferdos J, Rahman MM, Jesmin SS, Rahman MA, Sasagawa T. Association between intimate partner violence during pregnancy and maternal pregnancy complications among recently delivered women in Bangladesh. Aggressive behavior. 2018;44(3):294–305. 10.1002/ab.21752 29417590

[35] Ferdos J, Rahman MM. Maternal experience of intimate partner violence and low birth weight of children: A hospital-based study in Bangladesh. Plos one. 2017;12(10). 10.1371/journal.pone.0187138 PMC5658163 29073222

[36] Li Y, Zheng Y, Qian J, Chen X, Shen Z, Tao L, et al Preventive effects of zinc against psychological stress-induced iron dyshomeostasis, erythropoiesis inhibition, and oxidative stress status in rats. Biological trace element research. 2012;147(1–3):285–91. 10.1007/s12011-011-9319-z 22274754

[37] Hassan ZA, Chelebi NA, BazzazandAA BS. Correlation of psychological stress to severity of anemia in Al-Haweeja women. European journal of pharmaceutical and medical Research. 2016;3:248–51.

[38] Eng S, Li Y, Mulsow M, Fischer J. Domestic violence against women in Cambodia: Husband’s control, frequency of spousal discussion, and domestic violence reported by Cambodian women. Journal of Family Violence. 2010;25(3):237–46.

[39] Antai D. Controlling behavior, power relations within intimate relationships and intimate partner physical and sexual violence against women in Nigeria. BMC public health. 2011;11(1):511. 21714854 10.1186/1471-2458-11-511 PMC3161889

[40] Finkelstein J, Yates JK. Traumatic symptomatology in children who witness marital violence. International journal of emergency mental health. 2001;3(2):107–14. 11508563

[41] Goli S, Rana MJ, Gouda J. Intimate Partner Violence: effects on maternity care and pregnancy outcomes in India. Economic & Political Weekly, 2020:55(6), 71.

